# Beverage Intake and Its Effect on Body Weight Status among WIC Preschool-Age Children

**DOI:** 10.1155/2019/3032457

**Published:** 2019-01-16

**Authors:** Andrea Charvet, Fatma G. Huffman

**Affiliations:** Department of Dietetics and Nutrition, Florida International University, Miami 33199, USA

## Abstract

Given the prevalence and consequences of childhood obesity, efforts are being made to identify risk factors and design evidence-based interventions to reduce its impact. Food and beverage consumption habits are established early in life, making preschool-age children an important group to focus on. This cross-sectional study explored beverage intake and its association with body weight status among low-income preschool-age children enrolled in the Special Supplementation Nutrition Program for Women, Infants, and Children (WIC). Authorized representatives for children between the ages of 3 and 4.9 years were interviewed at WIC clinics in Broward County, Florida. Anthropometric data were collected from the WIC data system. The intake of sugar-sweetened beverages (SSB), particularly fruit drinks, was significantly higher in overweight/obese children when compared with their under/normal weight counterparts. Independent of body weight status, the preschool-age children were consuming on average over twice as much as the recommended intake of 100% fruit juice per day for that age group. Close to 80% of the overweight/obese children consumed low-fat or fat-free milk most often than any other type of milk. The intake of SSB was positively correlated with both the intakes of 100% fruit juice and milk, and negatively correlated with the intake of water. When body weight status, race/ethnicity, and intake of other beverages were held constant, SSB intake was positively associated with milk intake and negatively associated with water intake. Results from this study support the need to encourage water intake and discourage SSB intake in an effort to reduce the risk for overweight and obesity in WIC-participating preschool-age children. Emphasizing the need to follow the recommendation to limit 100% fruit juice intake to 4 to 6 oz. per day is important when counseling families with young children. Efforts to increase awareness of the health consequences associated with consuming high-fat milk should continue.

## 1. Introduction

Childhood obesity is a serious concern. Children from racial and ethnic minorities and those from low-income families are disproportionally affected. The latest data on obesity prevalence in children ages 2 to 5 years from the National Health and Nutrition Examination Surveys (NHANES) 2011–2014 show an overall rate of 8.9% [1]. Obesity prevalence rate for Hispanics was 15.6%, for non-Hispanic Blacks was 10.4%, for non-Hispanic Whites was 5.2%, and for non-Hispanic Asians was 5.0%. Obesity surveillance in young children from low-income families is being conducted through collaboration between the United States Department of Agriculture (USDA) and the Centers for Disease Control and Prevention (CDC) using data from the Special Supplementation Nutrition Program for Women, Infants, and Children (WIC) participants and program characteristics. Overall obesity prevalence among WIC participants ages 2 to 4.9 years was 14.5% in 2014 [2], substantially higher than the overall national prevalence of obesity.

WIC provides supplemental food, nutrition education, and referrals to community services for low-income, nutritionally at risk women, infants, and children up to 5 years of age. Race data from 2014 show that Whites are the largest group of WIC participants (58.7%), followed by African Americans (20.3%), American Indians or Alaska Natives (11.1%), and Asian or Pacific Islanders (4.1%); Hispanics accounted for 41.6% of all participants [3]. On average, more than a quarter of all children younger than 5 years of age in the United States participate in WIC [4]. Despite the substantial participation of young children in the WIC program, limited research has been conducted to evaluate the impact of beverage intake on body weight among this population.

Multiple risk factors are associated with childhood obesity. Current research on the relationship between beverage intake and body weight in children focuses mainly on sugar-sweetened beverages (SSB) intake, and to a lesser extent on juice and milk intake. SSB include sodas, fruit drinks (including lemonade, flavored water, and fruit nectars with added sugars), commercially prepared teas and coffees, and sports and energy drinks. SSB intake has been positively associated with the risk for overweight and obesity [5–8]. Because SSB can contribute excess calories while providing few or no key nutrients, the 2015–2020 Dietary Guidelines for Americans recommends the intake to be within the overall calorie limit for addead sugars, which is less than ten percent of total daily calories [9]. According to NHANES 2011–2014 data, U.S. children ages 2 to 19 years consumed on average 143 kcal/day from SSB, corresponding to 7.3% of their daily energy intake [10]. The intake of 100% fruit juice among U.S. children ages 2 to 18 has been increasing for the past few decades [11, 12]. One hundred percent fruit juice intake is associated with an increased nutrient adequacy in children's diets and may play a beneficial role when combined with a healthy dietary pattern [13, 14]. Despite this, an elevated intake may contribute to excessive calories and increase the risk for obesity. The American Academy of Pediatrics recommends at most 4 oz. of 100% fruit juice per day for toddlers ages 1 to 3 and 4 to 6 oz. for children ages 4 to 6 [15]. Results of studies on the relationship between 100% fruit juice intake and body weight are not conclusive. On the other hand, there is enough evidence to support that reducing the intake of SSB will help reduce obesity prevalence in both children and adults. Nevertheless, studies including preschool-age children are in a much smaller number and data from WIC participants are insufficient and limited to Hispanic toddlers [16, 17]. It has been suggested that potentially WIC may play a role in the decline of SSB consumption among young children and in the reduction of fruit juice consumption through the revisions of WIC food packages [18].

There is no consensus on the association between milk intake and the risk for overweight or obesity. The USDA 2015–2020 Dietary Guidelines recommends that preschool-age children consume 2 to 2 1/2 cups of low-fat milk per day [9]. Studies including children found mostly an inverse association between milk consumption and adiposity, possibly due to the protective role of dietary calcium [19, 20]. Barba et al. [21] demonstrated a significant inverse association between frequency of milk intake and body mass in a large sample of elementary school children in Italy. Other studies have found higher milk consumption by children to be associated with higher body mass [22], or have found no relationship between milk intake and body weight status [23]. It has been suggested that replacing SSB with milk may reduce weight gain in children [24, 25].

Food preferences and eating patterns are developed during early childhood and followed through adulthood [26, 27], highlighting the importance of understanding the risk factors for childhood obesity at an early age, particularly in at-risk populations. This study aimed to explore beverage intake and its relationship with body weight in a sample of children ages 3 to 4.9 years participating in the WIC Program in Broward County, Florida.

## 2. Methods

This cross-sectional study included a convenience sample collected from all seven Broward County Health Department WIC clinics. Broward County WIC is the second largest agency in Florida, having served more than 48 thousand clients during the 2017 fiscal year [28]. Inclusion criteria consisted of being between the ages of 3 and 4.9 years, currently enrolled in the WIC program in Broward County, being present at one of the WIC clinics for a certification or mid-certification visit, and having height and weight data less than 30 days old updated on the WIC data system. Exclusion criteria were any medical health condition, as indicated by medical nutritional risks by WIC codes, children under foster care, legal guardians who did not know the biological mother's pregnancy history, children accompanied by an authorized representative who did not participate on the daily care of the child, and children accompanied by an authorized representative who could not communicate in English or Spanish.

Recruitment flyers were placed at participating Broward County clinics. Authorized representatives for children who would qualify for the study were approached by the research staff while waiting to receive their WIC services and invited to participate in the study. If interested, the authorized representative for the child met the researcher in an available location inside the WIC clinic to participate in the interview after they finished with their WIC appointment services. Informed consent was obtained from the authorized representative, allowing the interview to be conducted and providing permission to access the child's data from the WIC data system. The authorized representative for each participant received a $10 gift card from a local grocery store to compensate for the time they spent to participate.

Approval to conduct the study was obtained from the Florida International University Institutional Review Board (IRB) and the State of Florida Department of Health IRB. Study protocol included secondary analysis of data accessed through the WIC data system and the use of a questionnaire to collect additional data not available through the WIC data system. Research Electronic Data Capture (REDCap), a secure web application for building and managing surveys and databases, was used for data collection and data management.

**Figure 1:**
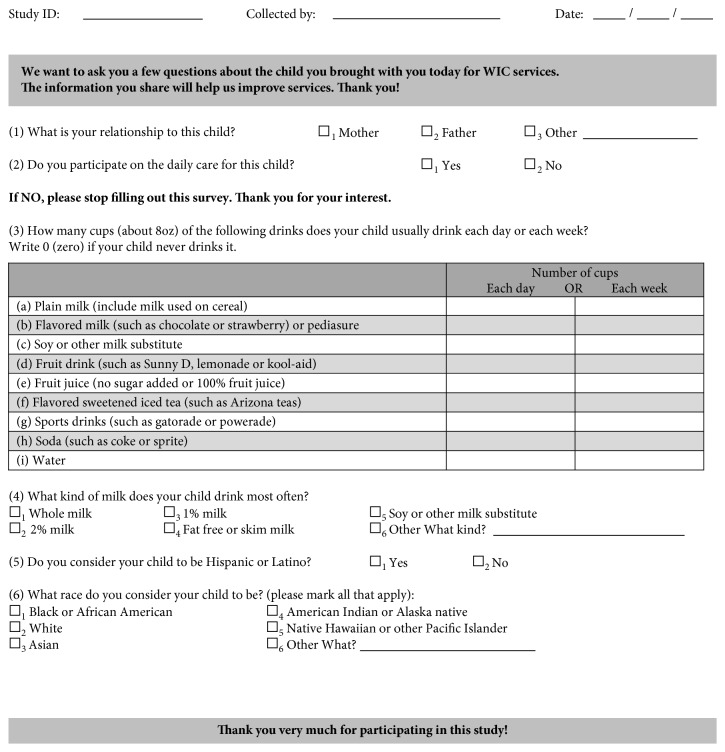
Questions from the larger instrument used for data collection included in this study.

The data exploring beverage intake analyzed in this study were collected using a larger questionnaire adapted from a previously validated instrument used by Nelson et al. [29] in a WIC population. The questionnaire was validated for use in this WIC population in a pilot test before the initiation of data collection. Data collected included amounts of different beverages (in number of 8 ounces cups) consumed per day, or per week if the beverage is not consumed by a child daily; type of milk most often consumed; and race and ethnicity (Figure 1). The larger questionnaire also collected additional data, which were not pertinent to this paper. Data fields collected from the WIC data system included the following: age, gender, anthropometric data (height, weight, BMI, BMI-for-age, weight-for-age, height-for-age, and weight-for-length), and biochemical data (hemoglobin and/or hematocrit). Crespi et al. [30] conducted a study to assess the accuracy of anthropometric measurements collected by WIC staff. They compared the WIC data with the “gold standard” measurements collected by trained research personnel at seven clinics in California (*n*=287) and concluded that height and weight of children ages 2–5 years collected by trained WIC staff were sufficiently accurate to be used for monitoring and research purposes. Definition of underweight, overweight, and obesity was based on the CDC [31] guidelines for BMI-for-age weight status categories and the corresponding percentiles: underweight (less than the 5^th^ percentile), healthy weight (5^th^ percentile to less than the 85^th^ percentile), overweight (85^th^ percentile to less than the 95^th^ percentile), and obese (equal to or greater than the 95^th^ percentile).

### 2.1. Data Analysis

Characteristics of the study population are presented using descriptive statistics. For data analysis purposes, the child's body weight category was combined into 2 groups, under/normal weight and overweight/obese. Average daily intake of beverages was calculated by multiplying the number of days a child drank that beverage per week by how many ounces per day and dividing by 7 days on a week. Sugar-sweetened beverage intake is an average of the daily intake of fruit drink, sweet tea, sports drink, and soda. The kind of milk the child consumed most often was grouped into whole and 2% milk, 1% and fat-free milk, and soy or other type.

For data analyses and to increase comparability with other studies, race and ethnicity were combined as one variable, race/ethnicity. Because close to 94% of our sample was composed of either Hispanics/Latinos or non-Hispanic Blacks/African Americans, for dichotomization purposes, in some analyses, only these two race/ethnicities were included. Independent samples *t*-tests were conducted to test for the relationship between the mean intake of different beverages and the child's body weight category combined, race/ethnicity, gender, age, and hemoglobin status. The relationship between the intakes of different beverages was tested using Pearson's correlation. Chi-square test of homogeneity was used to compare the type of milk consumed by the child's body weight category combined. Variables found to be significantly correlated with the intake of SSB were included in a multiple regression model. The effect of confounders including body weight category, race/ethnicity and the intake of 100% fruit juice, milk, and water on the intake of SSB was analyzed.

All calculations were carried out with SPSS software version 24 (SPSS In. Chicago, IL). A significance level of *p* < 0.05 was used for all statistical tests.

## 3. Results

From the 200 subjects who consented to participate in the study 3 extreme outliers, who reported more than 10 eating episodes in one day, were excluded. Most surveys have been answered by mothers (92.9%), with some by fathers (4.1%) and by grandmothers (3%). A total of 197 children were included in the data analysis. Children were between ages 3.0 to 4.9 years; mean age was 3.9 years (SD=0.6). Descriptive statistics are presented in Table 1. It is important to note that in our sample 86.5% of the Hispanic or Latino children were Whites, and 87.1% of the non-Hispanic or Latino children were Blacks or African Americans.

**Figure 2:**
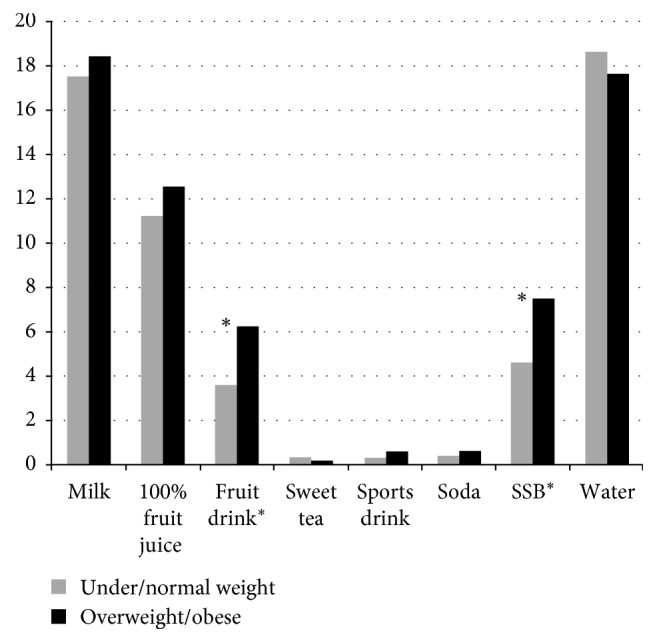
Daily average beverage intake in ounces, by weight status. SSB include fruit drink, sweet tea, sports drink, and soda. ^*∗*^Independent samples *t*-test significant at *p* < 0.05.

Children consumed on average 17.8 oz. of milk, 11.6 oz. of 100% fruit juice, 5.5 oz. of SSB, and 18.2 oz. of water per day. Beverage intake by weight status is reported on Figure 2. The intake of SSB including fruit drinks was significantly higher in overweight/obese children when compared to normal/underweight children (*p*=0.024). The intake of SSB was more than 2 times greater in non-Hispanic Blacks than in Hispanics/Latinos. Non-Hispanic Blacks also consumed significantly more 100% fruit juice. Beverage intake by race/ethnicity is reported on Table 2.

There was no significant difference in beverage intake by gender or age. Children with hemoglobin levels within normal limits drank significantly more SSB than children with low hemoglobin (5.91 ± 7.67 oz/day vs. 3.14 ± 5.45 oz/day, *p*=0.034). There was no significant difference in hemoglobin levels by any other beverage intake, including total milk intake (data not reported). The intake of SSB was positively correlated with both the intakes of 100% fruit juice (*r*=0.219, *p*=0.002) and milk (*r*=0.211, *p*=0.003), and negatively correlated with the intake of water (*r*=−0.231, *p*=0.001). All correlations were considered small but significant. Overall, 65.5% of the participating preschool-age children consumed 1% or fat-free milk most often than any other type of milk, being the first choice mostly for overweight/obese children (Table 3).

A multiple regression was run to predict SSB intake from body weight category, race/ethnicity, 100% fruit juice intake, milk intake, and water intake. The multiple regression model statistically significantly predicted SSB intake, *p* < 0.001. *R*^2^ for the overall model was 17.1%, with an adjusted *R*^2^ of 14.9%, indicating a small size effect. All variables except 100% fruit juice intake added significantly to the prediction. Predicted average SSB intake per day was 2.74 oz. less for under/normal weight children than that predicted for overweight/obese children. For every ounce in water intake increase, SBB intake decreased by 0.175 oz. Results from the multiple regression are presented in Table 4.

## 4. Discussion

We found a significantly greater intake of SSB in overweight/obese WIC participating preschool-age children when compared to under/normal weight counterparts. This was mostly due to a greater intake of fruit drinks. Overweight/obese children presented an intake of fruit drinks per day over 72% greater than under/normal weight children. Our finding is in agreement with findings from a recent review that reports consistent evidence that SSB increase the risk for overweight/obesity among children and adolescents [8]. There was no significant difference in 100% fruit juice intake by weight category. A recent study including a nationally representative sample of 2- to 5-year-old children found no difference in the prevalence of overweight/obesity between consistent 100% fruit juice drinkers and inconsistent or nondrinkers using cross-sectional analysis [32]. The same study, which did not adjust for energy intake, did find that children who drank 100% fruit juice regularly at age 2 years had greater increases in BMI *z*-score by age 4 years than those who did not drink juice frequently or did not drink it at all [32]. Studies controlling for energy intake found no relationship between 100% fruit juice consumption and the prevalence of overweight or obesity in children [14]. Because of the nature of the design of this study, energy intake was not measured.

The 2010 Dietary Guidelines Advisory Committee Report [33] includes a greatly reduced intake of SSB and smaller amounts of fruit juice as factors associated with preventing excess adiposity in children. Research has shown that SSB consumption is the lowest among 2- to 5-year-old children, increasing with age [10]. The preschool-age children in our study consumed more 100% fruit juice than SSB. On average, we found that children were consuming 11.6 oz. of fruit juice and 5.5 oz. of SSB per day. Nevertheless, the average 100% juice intake was twice as much as the American Academy of Pediatrics recommendation, with a few children reporting a daily intake up to 6 times greater than the recommendation. Despite the beneficial nutrient intake associated with 100% fruit juice [13], the excessive intake of any food may lead to weight gain. National efforts have been made to support a limited exposure of high obesity risk preschool-age children to quickly ingested, energy-dense beverages, including fruit juice [34]. Children ages 1 to less than 5 receive a total of 128 oz. of 100% fruit juice per month in the WIC food package, which is equivalent to approximately 4 ounces per day. Early in 2017, the National Academies of Science, Engineering, and Medicine provided specific recommendations for a review of WIC food packages, including the substitution of a cash value voucher for fruits and vegetables in lieu of juice [35]. There already are no SSB in the WIC food package. Recommended amounts for juice intake should be reinforced when providing it to children.

Daily average intake of milk was not significantly different by weight category. Studies including children found mostly an inverse association between milk consumption and adiposity, possibly due to the protective role of dietary calcium [19, 20]. A significantly lower prevalence of overweight was reported in children consuming whole milk daily when compared with those who consumed milk less frequently [21]. Since the revisions in WIC food packages implemented in all states by 2009 there has been an improvement in the healthfulness of foods and mostly beverages purchased by WIC participants [36]. Since then WIC provides 1% milk to all participants 2 years of age and older who are not underweight or at risk of being underweight, in an attempt to reduce saturated fat and cholesterol intake. A study conducted in the New York State WIC program found an increased consumption of low-fat or fat-free milk by 4.5% among children 2–4 years after the implementation of the revised food package [37]. Nevertheless, 29.4% of the WIC participating preschool-age children in our study consumed 2% or whole milk, suggesting the importance of nutrition education focusing on the risks of providing high-fat milk to children older than the age of 2 years. Contrary to what we expected, high-fat milk intake was more prevalent in under/normal weight preschoolers. This difference remained significant if we considered the underweight children, who may choose to receive 2% milk in their WIC benefits package, independently of the normal weight children. This suggests a success of the nutrition education provided to authorized representatives of overweight or obese WIC preschool-age children approaching whole milk versus low-fat or fat-free milk.

Data from the 2017 US Census Bureau indicate that in South Florida only about 26% of the population is White non-Hispanic [38]. Current data provided by Broward County WIC administration indicate that 57.4% of the population served is Black or African American, 43.6% is White, 2.6% is Asian, 0.6% is American Indian or Alaska native, 0.5% is native Hawaiian or other Pacific Islander, and 4.6% is multiracial. As per the same data source, Hispanic or Latino represents 35.8% of the Broward County WIC population. This breakdown resembles the race and ethnicity breakdown from our sample. Our study is unique in the fact that it included low socioeconomic status families, with more than 93% of the participants being either non-Hispanic Blacks or Hispanics/Latinos, a population that is at increased risk for overweight and obesity. When comparing these two groups, Non-Hispanic Black children consumed a significantly greater amount of 100% fruit juice and SSB than Hispanic/Latino children, placing them at a greater risk for overweight and obesity. In a study including low-income African American preschool-age children, the authors found that this population had a greater intake of SSB than the overall population and that it was positively associated with BMI *z*-scores [39].

The intake of SSB was positively correlated with both the intakes of 100% fruit juice and milk, and negatively correlated with the intake of water. When body weight status, race/ethnicity, and intake of other beverages were held constant, SSB intake was positively associated with milk intake and negatively associated with water intake. Encouraging water intake has been suggested as a strategy to reduce SSB intake in young children [18]. A longitudinal study including 9-year-old children found that replacing SSB with equal amounts of water or milk, but not of 100% fruit juice, was associated with a significant decrease in BMI *z*-score, waist circumference, and sum of four skinfolds [24].

Results from this study add to the body of literature in childhood obesity by including WIC participating children, a population at a higher risk for overweight and obesity that is not often included in studies. One of the strengths of this study was the use of a researcher-administered questionnaire, providing virtually no unanswered questions and allowing the researcher to probe as necessary. Another strength is the reliance on measured and not self-reported anthropometric data. In addition, the inclusion of a sample consisted of WIC participants granted control for socioeconomic status. Our findings highlight the importance of providing nutrition education to WIC families aimed at increasing water intake, decreasing 100% fruit juice intake, and avoiding fruit drinks in an effort to reduce the risk for overweight and obesity in preschool-age children.

Among the limitations of this study are its cross-sectional design and the lack of data on energy and nutrient intake. Children who are consuming more SSB may also have a greater intake of other energy-dense foods, which may collaborate to the relationship we found between SSB intake and the prevalence of overweight and obesity. Estimating energy intake from beverages to further analyze the data would yield questionable results, since collecting data on the composition of food and beverages consumed by the participants was not one of the aims of this research. Considering that the intake of milk and 100% fruit juice was much larger than that of SSB but still not significantly associated with overweight/obesity, we may suggest that it was the effect of SSB intake that was driving the association with overweight/obesity and not the additive effect of different beverages. Despite this, we may not rule out the potential effect of energy intake from other sources as a confounding factor.

Another limitation, as with any self-reported food and beverage intake data, potential biases due to underreporting or overreporting of beverage intake may be present. Caution needs to be exercised when interpreting data on specific ounces of intake since errors may be present when reporting a specific measure. Furthermore, this study included a convenience sample and results are not generalizable, even though it presents a valid representation of the WIC population in South Florida. Longitudinal studies including a large, racially and ethnically diverse population from different socioeconomic levels may help elucidate the impact of different beverages intake on the risk for overweight and obesity in young children from different backgrounds. By gaining a better understanding, researchers will be equipped with the necessary tools to propose interventions targeted at specific populations. Additional research is needed on the effectiveness of different obesity prevention interventions with WIC clients.

## 5. Conclusion

Results from our study provide solid evidence of the importance of discouraging parents from offering SSB to their young children. Even though 100% fruit juice intake was not found to be significantly associated with body weight, the finding that preschool-age children were consuming over twice as much as the recommended daily intake of fruit juice highlights the importance of encouraging WIC families to follow the recommendation to limit their children's 100% fruit juice intake to no more than 4 to 6 oz. per day. Another important recommendation provided as part of the WIC nutrition education approaches the need to provide low-fat milk to children ages 2 years and older. Most of the participating children were already following that recommendation; however, close to 30% were not, suggesting the efforts need to continue. This study is of substantial importance since most of the participants were either Hispanics or African Americans, given the greater prevalence of overweight and obesity found among these populations.

## Figures and Tables

**Table 1:** Description of study sample (*N*=197).

Characteristic		Frequency % (*N*)
Ethnicity	Hispanic or Latino	52.8% (104)
	Non-Hispanic or Latino	47.2% (93)

Race	Black or African American	44.7% (88)
	White	48.2% (95)
	Asian	2.5% (5)
	American Indian or Alaska native	0 (0)
	Native Hawaiian or Pacific Islander	0 (0)
	Mixed race^a^	4.6% (9)

Race/ethnicity	Hispanic or Latino	52.8% (104)
	Non-Hispanic Black	41.1% (81)
	Non-Hispanic White	2.5% (5)
	Non-Hispanic Asian	2.5% (5)
	Other and mixed race^a^	1% (2)

Gender	Male	45.7% (90)
	Female	54.3% (107)

Weight category	Underweight	3.6% (7)
	Normal weight	64.4% (127)
	Overweight	16.8% (33)
	Obese	15.2% (30)

Hemoglobin status	Low	12.2% (24)
	Within normal limits	86.8% (171)

^a^Mixed race indicates more than one race has been selected.

**Table 2:** Daily average beverage intake in ounces, by race/ethnicity (mean ± SD).

Beverage	Race/ethnicity		
	Hispanic or Latino	Non-Hispanic Black	*p* value
Plain milk	10.9 ± 8.5	13.2 ± 10.3	0.099
Flavored milk	6.7 ± 8.4	5.0 ± 7.1	0.147
Milk (any)	17.6 ± 8.3	18.2 ± 10.4	0.624
Fruit juice	10.4 ± 8.3	13.5 ± 8.7	0.014^*∗*^
Fruit drink	2.5 ± 4.6	7.1 ± 8.1	<0.001^*∗*^
Sweet tea	0.1 ± 0.5	0.3 ± 1.1	0.109
Sports drink	0.3 ± 0.7	0.6 ± 1.9	0.102
Soda	0.4 ± 1.1	0.5 ± 1.7	0.607
SSB^a^	3.3 ± 5.1	8.6 ± 9.1	<0.001^*∗*^
Water	18.3 ± 8.5	17.6 ± 8.2	0.589

^a^SSB include fruit drink, sweet tea, sports drink, and soda. ^*∗*^Independent samples *t*-test significant at *p* < 0.05.

**Table 3:** Proportion of type of milk most often consumed by weight category combined in WIC participating preschool children.

Type of milk	Under/normal weight^*∗*^ (*n*=134) % (*N*)	Overweight/obese^*∗*^ (*n*=63) % (*N*)	Overall (*n*=197) % (*N*)
Whole or 2%	35.8 (48)	15.9 (10)	29.4 (58)
1% or fat-free	59.0 (79)	79.4 (50)	65.5 (129)
Soy or other	5.2 (7)	4.8 (3)	5.1 (10)

^*∗*^Chi-square test of homogeneity, *p*=0.014.

**Table 4:** Predictors of sugar-sweetened beverages intake (ounces) in WIC participating preschool-age children.

Predictor variable	*B* ± SE	*β*	*p* value
Body weight category^a^	−2.740 ± 1.061	−0.172	0.011^*∗*^
Race/ethnicity^b^	1.957 ± 0.659	0.198	0.003^*∗*^
Milk intake per day	0.136 ± 0.056	0.164	0.016^*∗*^
100% juice intake per day	0.096 ± 0.062	0.108	0.123
Water intake per day	−0.175 ± 0.060	−0.197	0.004^*∗*^

Dependent variable: SSB intake average per day (oz.).^a^ Under/normal weight compared with overweight/obese as the reference group.^b^ Non-Hispanic Black compared with Hispanic/Latino as the reference group. *B* = unstandardized regression coefficient; SE = standard error of the coefficient; *β* = standardized coefficient. ^*∗*^ Significant at *p* < 0.05.

## Data Availability

The authors can be contacted regarding inquiries to the data set and data sharing.
